# Zinc and Copper Ions Induce Aggregation of Human β-Crystallins

**DOI:** 10.3390/molecules27092970

**Published:** 2022-05-06

**Authors:** Vanesa Ramirez-Bello, Javier Martinez-Seoane, Arline Fernández-Silva, Carlos Amero

**Affiliations:** LABRMN, Centro de Investigaciones Químicas, Instituto de Investigación en Ciencias Básicas y Aplicadas, Universidad Autónoma del Estado de Morelos, Cuernavaca 62209, Mexico; vanesa.ramirezbel@uaem.edu.mx (V.R.-B.); javier.martinezs@uaem.edu.mx (J.M.-S.); arline.fernandezsil@uaem.edu.mx (A.F.-S.)

**Keywords:** crystallins, human beta crystallins, copper, zinc, cataracts

## Abstract

Cataracts are defined as the clouding of the lens due to the formation of insoluble protein aggregates. Metal ions exposure has been recognized as a risk factor in the cataract formation process. The γ and β crystallins are members of a larger family and share several structural features. Several studies have shown that copper and zinc ions induce the formation of γ-crystallins aggregates. However, the interaction of metal ions with β-crystallins, some of the most abundant crystallins in the lens, has not been explored until now. Here, we evaluate the effect of Cu(II) and Zn(II) ions on the aggregation of HβA1, as a representative of the acidic form, and HβB2, as a representative of the basic β-crystallins. We used several biophysical techniques and computational methods to show that Cu(II) and Zn(II) induce aggregation following different pathways. Both metal ions destabilize the proteins and impact protein folding. Copper induced a small conformational change in HβA1, leading to high-molecular-weight light-scattering aggregates, while zinc is more aggressive towards HβB2 and induces a larger conformational change. Our work provides information on the mechanisms of metal-induced aggregation of β-crystallins.

## 1. Introduction

Cataracts remain one of the leading causes of blindness worldwide, affecting more than 90 million people [1,2,3]. There are several risk factors associated with cataract formation, such as UV radiation, smoking, and diabetes, among others [4,5,6]. In recent years, metal ion exposure has been recognized as a risk factor, capable of causing protein aggregation. Even though the concentration of metals in the lens is usually low, in the range of 0.4 and 30 μg of metal/g of lens tissue [7,8], this concentration increases in eyes with cataracts [9,10,11,12].

Crystallins are the main proteins in the human lens and are divided into two superfamilies: α-crystallins and βγ-crystallins [13,14,15]. The α-crystallins are members of the small heat-shock protein (sHsp) and function as chaperons. The βγ-crystallins are the main structural proteins of the lens and share a similar fold in their tertiary structures composed of two domains connected by a linker peptide, each one with two Greek-key motifs. The γ-crystallins have been described as monomers, while the β-crystallins can form dimers or larger oligomers [13,14,16].

The β-crystallins constitute ~40% of the crystallins of the lens and historically are classified as acidic (βA) or basic (βB) [14,17]. The acidic isoforms have four members (βA1, βA2, βA3, βA4) while the basic ones contain three isoforms (βB1, βB2, βB3) [18]. These proteins have N-terminal or C-terminal extensions of different lengths with respect to the γ-crystallins [19]. The acidic isoforms possess only N-terminal extensions while the basic crystallins also have C-terminal extensions. Generally, the β-crystallins are more widely expressed, leading to a spatial distribution that encompasses both the nucleus and the cortex of the lens [20]. Two of the isoforms, βA1 and βA3, are produced from the same gene, Cryba1, by leaky ribosomal scanning [21]. Human βB2-crystallin is one of the most abundant β-crystallins in the developed lens [22] and constitutes 14–20% of the total lens protein [23].

To date, three-dimensional structures have been reported for βA4, βB1, βB2, and βB3 [24,25]. HβB2 has been crystallized as a domain-swapped dimer [25,26]; however, solution studies have suggested that dimerization occurs through a compact face-en-face dimer [27]. Moreover, the stability of the βB2 dimers studied by computational methods revealed that the optimal configuration of the protein was face-en-face and not domain swapping [28].

Several studies have shown that copper and zinc induce the formation of aggregates of different γ-crystallins by interacting in more than one binding site for each of the metal ions [29,30,31,32,33,34]. Recently, we demonstrated that Cu(II) and Zn(II) bind at least at two different sites and induce aggregation of human γD-crystallin following different pathways. Zn(II) ions produce a small conformational rearrangement and aggregate through metal bridging without any unfolded intermediate, whereas Cu(II)-induced aggregation includes a lag time in which the N-terminal domain partially unfolds while the C-terminal domain and parts of the N-terminal domain remain in a nativelike conformation [33]. Nevertheless, the effect of metal ions on the β-crystallins has remained unexplored.

In this study, we evaluated the effect of Cu(II) and Zn(II) ions on the aggregation of HβA1, one of the acidic β-crystallins, and HβB2, a representative of the basic form, to better understand the metal-induced cataract formation mechanism. We used several biophysical techniques, such as turbidimetry, dynamic light scattering (DLS), fluorescence, infrared spectroscopy, and computational methods. We found that both metals induce a conformational change in the proteins, leading to high-molecular-weight light-scattering aggregates. The metal ions interact in a specific manner and induce different aggregation mechanisms. Our work provides information on the metal-induced aggregation of β-crystallins, showing that the diverse sequence of crystallins is consistent with multiple pathways for each protein and distinct processes for the different factors that affect them.

## 2. Results

Metal ions have emerged as a potential etiological agent for cataract formation. Cu(II) and Zn(II) have been reported to induce aggregation of various γ-crystallins. Here, we study the effect that these metal ions have on one member of the acidic β-crystallin and one member of the basic β-crystallin.

### 2.1. Bioinformatics and Molecular Modeling

**Figure 1:**
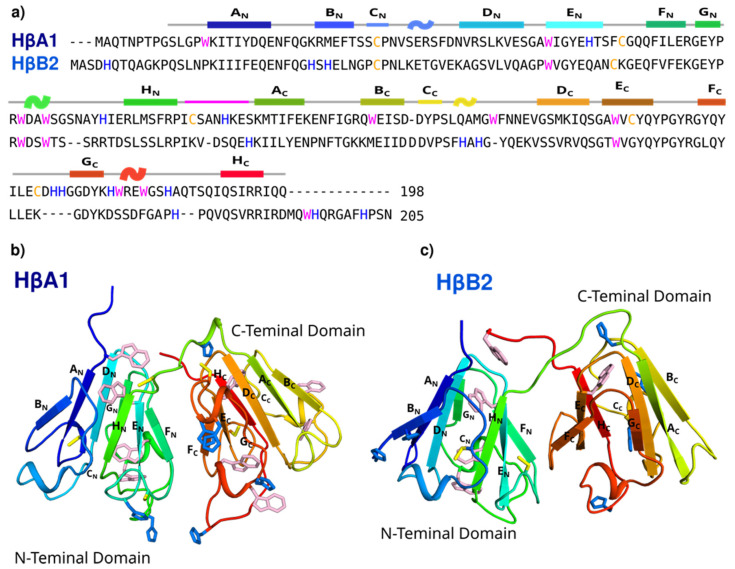
β-crystallin structures and sequences. (**a**) Sequence alignment of HβA1 and HβB2 crystallins. The elements of the secondary structure of the N-terminal and C-terminal domains and the linker region are shown. Histidine residues are shown in blue, cysteines in yellow, and tryptophan in magenta. Three-dimensional structure models of (**b**) HβA1 and (**c**) HβB2. Each strand is named A through H for each domain.

All βγ-crystallins share a similar three-dimensional structure, comprising two double Greek key domains; however, they differ in the detail of the sequence and the length of the extensions at the terminals. HβA1 is a 198 residue protein with an isoelectric point of 6.4 [14], while HβB2 is composed of 205 residues and has an isoelectric point of 6.5 [14], and both sequences share 46.1% identity (Figure 1a). Both proteins are flanked by a small N-terminal extension, but only HβB2 contains a short C-terminal extension.

We made a homology model for HβA1, using HβA4 (PDB ID:3LWK) as a template. The first 9 residues were not included in the modeling. The structure of HβB2 was taken from PDB ID:1YTQ and modeled as a monomer (Figure 1b). The first 14 residues and the last 10 residues do not contain electron density in the crystal structure, and therefore were not included in the model. To perform the subsequent analyses, and because the β-crystallins have been described as dimers in solution, we built ‘face-en-face’ dimer models (Figure S1).

Sequence alignment of the β/γ-crystallins revealed that, although several features are conserved in the proteins, the reported binding sites for γ C, D, and S are not conserved in the β-crystallins (Figure S2). Histidines and cysteine residues are the most common binding residues for copper and zinc, with an observed prevalence of ~50% Cys and ~30% His for zinc and ~60% His and ~10% Cys for copper, followed by Glu and Asp residues [35]. HβA1 contains 7 His and 5 Cys, with one CXXXH motif on the linker. HβB2 contains 9 His and 2 Cys, with 3 His residues in the nonmodeled regions. Interestingly, there are several sites in the structure where the His and the Cys cluster together, making potential metal binding sites.

We then used MIB [36] and BioMetAll [37] metal binding prediction software to estimate the possible binding sites for Zn(II) and Cu(II). MIB searches for sequence similarities between the target protein and previously reported metal-binding site proteins, while BioMetAll looks for characteristic sequences in the protein backbone. As it can be observed in Figure S3, both programs predicted several possible binding sites for the two proteins; nevertheless, these binding regions are not the same. Considering the consensus result of the two predictors, there is a higher prevalence in the C-terminal domain for HβA1 and a higher prevalence in the N-terminal domain for HβB2.

### 2.2. Cu(II) and Zn(II) Ions Induce Formation of Light-Scattering Aggregates

**Figure 2:**
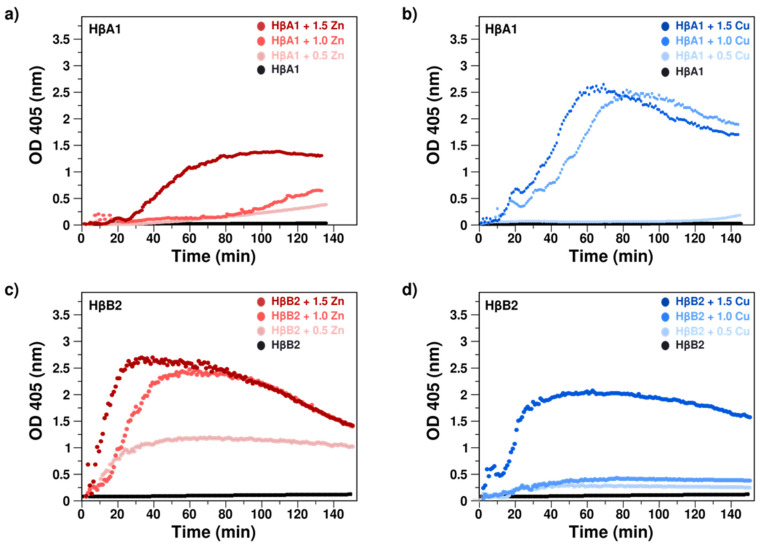
Effect of Zn(II) and Cu(II) as reported by turbidity assays at 37 °C. Absorbance at 405 nm as function of time for (**a**) HβA1 in the absence (black) and presence of 0.5, 1, and 1.5 equivalents of Zn(II) (red gradient); (**b**) HβA1 in the absence (black) and presence of 0.5, 1, and 1.5 equivalents of Cu(II) (blue gradient); (**c**) HβB2 in the absence (black) and presence of 0.5, 1, and 1.5 equivalents of Zn(II) (red gradient); (**d**) HβB2 in the absence (black) and presence of 0.5, 1, and 1.5 equivalents of Cu(II) (blue gradient). The change in absorbance is due to the formation of aggregates that scatter the light.

To assess the effect of Cu(II) and Zn(II) ions on the proteins, we monitored the optical density (OD) at 405 nm as a function of time with and without the metals at 37 °C. The formation of protein aggregates that scatter light would increase the absorbance of the solution. In the absence of metal ions, the absorbance of HβA1 and HβB2 remains almost unchanged with a net change of less than 0.1 OD after 12 h for both proteins, indicating that both proteins do not aggregate during the experiment time (Figure 2). Absorption spectra at different time points are shown in Figure S4.

Then, the effect of Zn(II) and Cu(II) on the β-crystallins was evaluated; after the addition of increasing amounts of metal ion equivalents (0.5, 1, and 1.5 eq), we observed severe changes in the absorbance (Figure 2).

For HβA1, the addition of increasing amounts of Zn(II) produces a steady increase in turbidity, indicating interaction with the metal ion. This interaction induces the formation of light-scattering aggregates. With 1.5 eq of metal ion, in which presumably the metal should be interacting in at least two different binding sites, we observed that the turbidity reaches a plateau (Figure 2). Visual inspection of the cuvette after the experiments shows large amounts of precipitation in the sample. Higher concentrations of metal ions produces severe precipitation in the cuvette, presumably due to the formation of higher molecular weight aggregates that precipitate. It should be noted that the values obtained are greater than 1, which implies that we are outside the linear zone, and it would not be possible to make any kinetic estimation.

The effect of Cu(II) on HβA1 was more pronounced, presenting a drastic increase in turbidity even for one metal ion equivalent. After longer times, we observed a decrease in turbidimetry that corresponds to the precipitation of large aggregates in the sample cuvette.

Interestingly, for HβB2, the effect of Zn(II) was more drastic than the effect observed on HβA1. Zinc induces an immediate increase in turbidity, reaching a maximum followed by a decrease. This decrease corresponds to the appearance of precipitated aggregates. The addition of increasing amounts of Cu(II) (0.5, 1, and 1.5 eq) also induces an increase in turbidity for the different concentrations, although the increase was more subtle.

Therefore, the effect observed for the crystallins HβA1 and HβB2 was the opposite; HβA1 appeared to be more affected by copper, while HβB2 was more sensitive to zinc. Thus, Cu(II) and Zn(II) exert different and specific effects on the aggregation of different Hβ crystallins.

To evaluate whether metal ion coordination forms intermolecular aggregates through metal ion bridging, we used EDTA as a metal chelator. Table 1 shows the soluble protein recovered after addition of excess EDTA. For both proteins with Cu(II) and Zn(II) ions, the addition of EDTA reduced the light-scattering intensity, increasing the amount of recovered protein. This indicates that at least some of the high molecular weight aggregates are due to metal bridging.

Overall, these results show that either zinc or copper induce light-scattering aggregation at 37 °C in HβA1 and HβB2. The difference in the shape of the spectra indicates that the metal–protein interactions for Zn(II) and Cu(II) are different. Furthermore, the fact that the metal-induced aggregation rate increases with concentration suggests that there is more than one binding site, consistent with what has already been observed with the γ-crystallins. Here, we are interested in the effect of the binding on at least two different sites; therefore, we decided to continue the experiments with 1.5 equivalents of the metals.

Metal-induced aggregates were further analyzed by dynamic light scattering (DLS) to measure changes in sizes induced by the ions. As a reference, we calculated the hydrodynamic translational diffusion coefficient predicted by the Hullrad [38] software from the face-en-face dimer models. It should be mentioned that the structural models do not include the extension at the beginning of the N-terminal domain or at the end of the C-terminal domain. The hydrodynamic radius (R_H_) and the translation diffusion coefficients obtained were R_H_~2.7 nm and D~7.9 × 10^−7^ cm^2^/s for HβA1 and R_H_~2.6 nm and D~8.1 × 10^−7^ cm^2^/s for HβB2.

**Figure 3:**
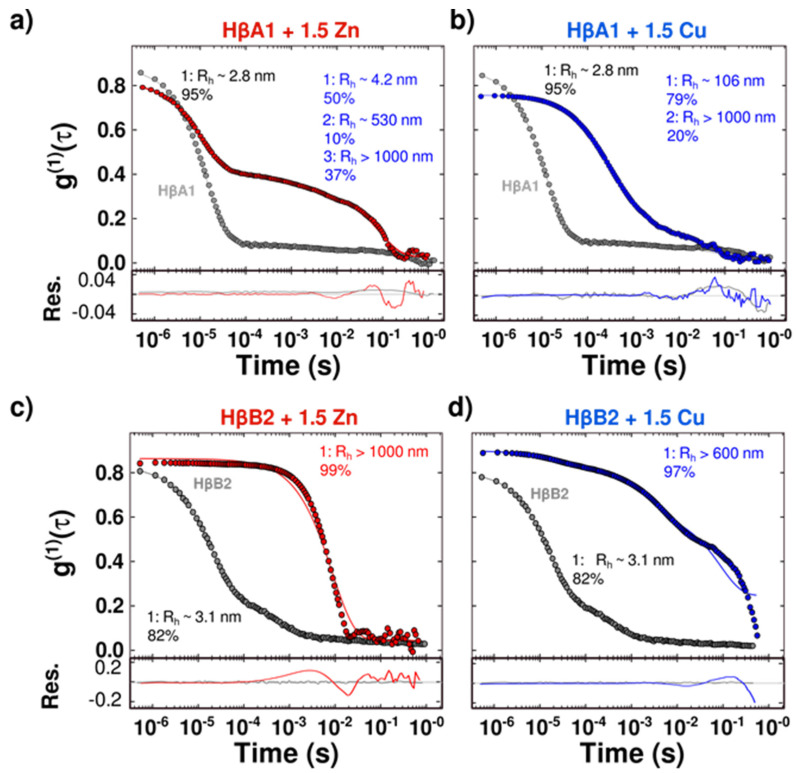
Protein oligomerization induced by metal-binding. (**a**) HβA1 correlation coefficient in the absence (gray) and presence of 1.5 equivalents of Zn(II) (red). (**b**) HβA1 correlation coefficient in the absence (gray) and presence of 1.5 equivalents of Cu(II) (blue). (**c**) HβB2 correlation coefficient in the absence (gray) and presence of 1.5 equivalents of Zn(II) (red). (**d**) HβB2 correlation coefficient in the absence (gray) and presence of 1.5 equivalents of Cu(II) (blue). Measurements with metal ions yielded a shift to the right indicating an increase in size due to oligomerization. Calculated R_H_ are shown. (Replica spectra are shown in Figure S5).

DLS measurements of HβA1 and HβB2 in solution show a correlation curve consistent with a small protein; however, the shoulder at the end of the curve suggests the presence of a larger species (Figure 3 and Figure S5). The most abundant conformer, which accounts for at least more than 80% of the total signal, has an apparent R_H_ of ~2.8 nm for HβA1, while the value obtained for HβB2 was an R_H_ of ~3.1 nm (Figure 3 and Figure S5). These results suggest that under these conditions, HβA1 and HβB2 consist mostly of dimeric proteins.

When the proteins were incubated with 1.5 equivalents of Zn(II) or Cu(II) ions at 37 °C for 40 min, the correlation curves shifted to the right, consistent with an increase in size due to the oligomerization induced by metal binding (Figure 3 and Figure S5). In all cases, the results show the formation of large oligomers with radii from several hundred to thousands of nanometers. These results confirmed that the increase in size of the oligomers is, in fact, responsible for the turbidity of the solution.

In general, these data confirm that both Cu(II) and Zn(II) induce the formation of larger aggregates opaque to light and the loss of soluble protein with different mechanisms.

### 2.3. Cu(II) and Zn(II) Ions Induce Conformational Changes

Intrinsic fluorescence spectroscopy was used to detect changes in the local environment of the tryptophan residues, which would report structural changes induced by the metals binding.

HβA1 has nine tryptophans, while HβB2 contains five tryptophans (Figure 1). Interestingly, HβA1 has four Trps in the N-terminal domain, while HβB2 only has three, but one of the Trps at the C-terminal extension of HβB2, in the structural model, appears to be positioned in a similar region to the fourth Trp of HβA1, probably playing the same structural role.

The fluorescence of these tryptophans would be affected by the surrounding chemical environment, making them excellent reporters of the overall fold of the protein. For HβA1, the emission maximum for the intrinsic fluorescence of the native protein is around 344 ± 2 nm, while the maximum shifts to 359 ± 2 nm when the protein unfolds (Figure S6). On the other hand, the maximum emission for the intrinsic fluorescence of the native HβB2 is 333 ± 2 nm, while this maximum shifts to 350 ± 2 nm when the HβB2 is fully unfolded (Figure S6). Therefore, the change in maximum emission can be used to investigate the differences between the structural states.

**Figure 4:**
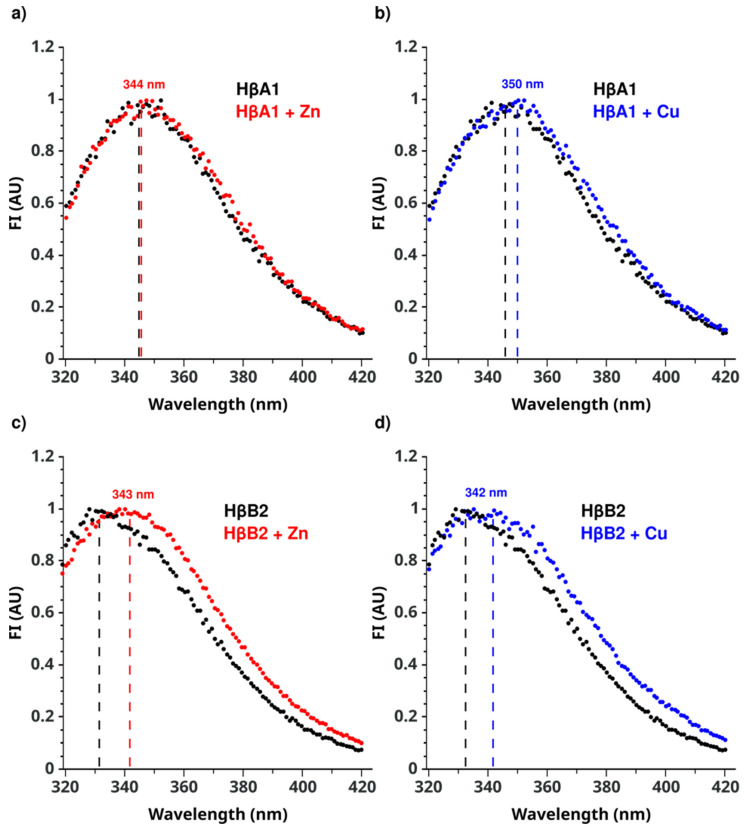
Normalized Intrinsic fluorescence. (**a**) HβA1 in the absence of metal ions (black) and in the presence of Zn(II) (red). (**b**) HβA1 in the absence of metal ions (black) and in the presence of Cu(II) (blue). (**c**) HβB2 in the absence of metal ions (black) and in the presence of Zn(II) (red). (**d**) HβB2 in the absence of metal ions (black) and in the presence of Cu(II) (blue). The emission spectrum was recorded in the range of 300 and 500 nm using an excitation wavelength of 295 nm at 37 °C. (Replica spectra are shown in Figure S7).

In the presence of 1.5 eq of Zn(II), we do not observe a shift in HβA1, indicating that there are no significant changes in the Trp environment upon metal binding. On the other hand, copper induces a small shift in the maximum from 344 ± 2 to 350 ± 2 nm, reporting a change in the Trp environment with the metal bound (Figure 4 and Figure S7). These results suggest that Zn(II) does not produce a change in the core of the protein while Cu(II) induces a small structural rearrangement that is responsible for the small change in fluorescence observed during the aggregation process.

For HβB2, the presence of 1.5 eq of Zn(II) induces a shift in the maximum from 333 ± 2 to 343 ± 2 nm (Figure 4 and Figure S7). A similar change was observed in the presence of Cu(II), with the maximum shifting from 333 ± 2 nm to 342 ± 2 nm after adding the metal. These data confirm that both Zn(II) and Cu(II) induce a structural rearrangement at the HβB2 core.

**Figure 5:**
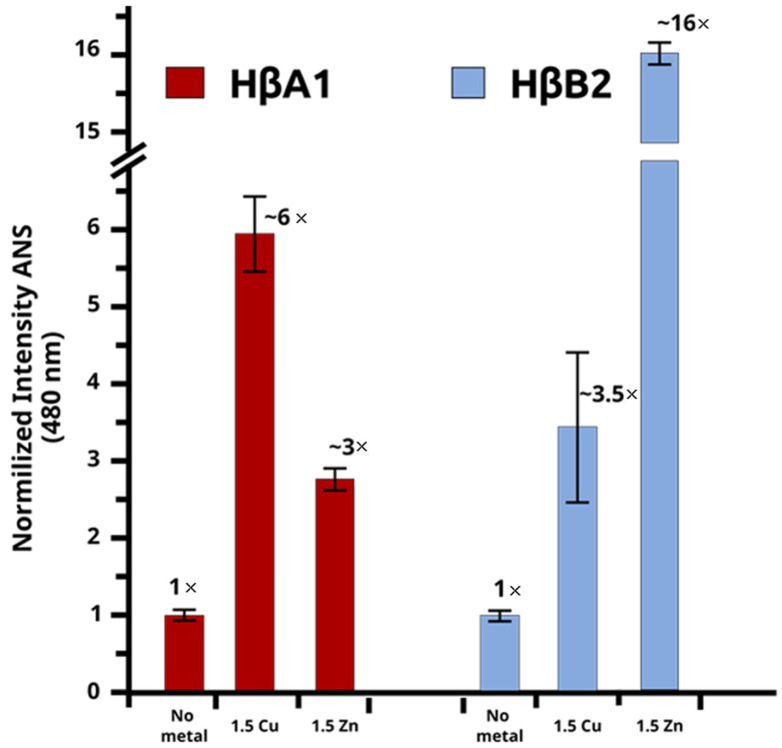
Normalized ANS fluorescence intensity. HβA1 (red) without the metals and with 1.5 eq of Cu(II) and Zn(II). HβB2 (blue) without the metals and with 1.5 eq of Cu(II) and Zn(II).

Then, we used ANS fluorescence as a probe of exposed hydrophobic surface area. An increase in the fluorescence intensity of ANS with respect to the folded protein would imply that ANS binds to new hydrophobic clusters formed upon metal binding, suggesting a partial unfolding of the crystallins. The dimer models for HβA1 and HβB2 present few exposed hydrophobic sites with the ability to bind to ANS molecules, consistent with the low ANS fluorescence obtained for the native proteins (Figure 5).

Contrary to the results obtained with Trp fluorescence, an increase in ANS fluorescence ~3 times greater than in the native protein for HβA1 with Zn(II) was observed. For Cu(II), the increase in ANS fluorescence was even larger, with ~6 times more intensity than in the native folded protein.

For HβB2, we observed a very large ANS fluorescence intensity in the presence of Zn(II), about ~12 times greater than the florescence of the native protein (Figure 5), indicating the exposure of large hydrophobic patches. In contrast, HβB2 in presence of Cu(II) shows a relatively small increase in ANS florescence (~3.5 times).

These results suggest that metal ions induce structural rearrangement that are responsible for the observed changes in the fluorescence during the aggregation process, confirming that the effect of 1.5 equivalents of Cu(II) or Zn(II) ions are different, thus indicating distinctive aggregation mechanisms.

We use infrared spectroscopy to characterize changes in secondary structure. The peaks obtained by deconvolution of the amide I band were assigned to specific types of secondary structure [39,40]. For HβA1, we distinguished a main peak corresponding to β sheet at 1634 ± 2 cm^−1^ and one peak at 1677 ± 2 cm^−1^ that could correspond to β turn or β sheet (Figure S8). We obtain two peaks (at 1588 ± 2 cm^−1^ and 1720 ± 2 cm^−1^) corresponding to absorbance of side chain. The fractions component corresponding to types of β-structure was ~88%. In addition, we obtained two small marginal bands (1604 cm^−1^ and 1592 cm^−1^) due to side-chain absorption.

With the addition of Zn and Cu, we obtained four similar peaks. The main peak was centered at 1635 ± 2 cm^−1^ and the second peak centered at 1678 ± 2 cm^−1^. The total fraction corresponding to β structures were ~92% for zinc and ~93% for copper, which suggests that the addition of metal ions does not significantly change the secondary structure of HβA1 (Figure S8).

For HβB2, we distinguished a main peak corresponding to β sheet at 1636 ± 2 cm^−1^ with 78% and two peaks that could correspond to β turn or β sheet high-frequency component at 1673 ± 2 cm^−1^ and at 1687 ± 2 cm^−1^ (Figure S8). We obtained also one peaks (at 1585 cm^−1^) corresponding to absorbance of side chain. The fractions component corresponding to types of β-structure was ~89%.

Again, with the addition of Zn(II) and Cu(II) we obtained four similar peaks. The main peak were centered at 1635 ± 2 cm^−1^ and the two peaks centered at 1669 ± 2 cm^−1^ and at 1683 ± 2 cm^−1^. Therefore, the total fractions corresponding to β structures were ~94% for zinc and ~95% for copper, indicating again that the interaction with metal ions does not significantly change the secondary structure (Figure S8).

We then used thioflavin T (THT) as a probe for amyloid fibrils formation; ThT exhibits strong fluorescence when it binds to amyloid fibrils. The metal-induced aggregates were not amyloid in nature, as confirmed by the assay (data not shown).

### 2.4. Changes in Stability Due to Metals

In an effort to account for their increased unfolding and aggregation propensity upon binding the metals, we examined the effect of Cu(II) and Zn(II) ions in the thermal stability of the β-crystallins. Denaturation experiments were performed from 37 to 90 °C using intrinsic Trp fluorescence and extrinsic ANS fluorescence.

Surprisingly, the Trp fluorescence spectra of HβA1 as a function of temperature did not show changes in the wavelength of maximum emission. This was probably due to the fact that we observed precipitation as soon as the temperature began to rise, suggesting the formation of large aggregates and the formation of precipitation, without the disruption of the core of the protein (Figure S9).

On the other hand, we observed HβA1 temperature unfolding curves when we measured the fluorescence of ANS. With the free protein, the fluorescence intensity of the ANS remains constant up to ~60 °C; then, we observed an increase in intensity indicating exposure of hydrophobic patches. The addition of Zn(II) and Cu(II) ions caused a drastic decrease in temperatures where the exposure of hydrophobic areas was observed (Figure S9), indicating a reduction in thermal stability due to metal ions.

The Trp fluorescence of HβB2 remains constant up to ~60 °C. A further increase in temperature shows an increase in the signal, suggesting a single transition from folded protein to unfolded protein. From the data, we calculated the middle point of thermal unfolding as 82.9 ± 0.3 °C. This value is consistent with previous reports and shows that the HβB2 is a highly stable protein.

Then, we performed the experiments in the presence of Cu(II) and Zn(II) ions as shown in Figure S9. The data shows a shift to the left in the spectra, indicating a decrease in the stability of the protein. Similar thermally unfolding spectra were observed in the presence of ANS, where also Zn(II) and Cu(II) ions caused a drastic increase in the hydrophobic areas exposed in the HβB2 crystallin.

Overall, these results show that the interaction of Zn(II) and Cu(II) ions with both β-crystallins decreases their thermal stability.

## 3. Discussion

Here, we study the effect of zinc and copper metal ions on the beta crystallins HβA1, as a representative of the acidic form, and HβB2, as a representative of the basic β-crystallins.

We used bioinformatics techniques to model the face-en-face dimer for both β-crystallins and predict potential metal binding sites. The alignments of the β with the γ-crystallins show that the residues previously proposed as residues interacting with Cu(II) and Zn(II) in the γ are not conserved in the β-crystallins. These residues are mostly His and Cys.

Nevertheless, several binding candidates were identified using the MIB and BioMetAll software. The interaction with more than one binding site is consistent with what has already been reported for the γ-crystallins; more than one metal ion is required to induce aggregation [33,34,41,42]. Furthermore, for the HγS crystallin, it was found that mutation of the binding residues resulted in more drastic aggregation; therefore, it was proposed that the first binding site serves as an oxidation sink [34]. For the HγD crystallin, a detailed aggregation pathway was described with the minimum concentration of 1.5 eq of metal [33].

Several biophysics techniques showed that in the absence of metal ions, HβA1 and HβB2 remain soluble, folded, and exhibit no tendency to aggregate, confirming that the free native protein does not change its light-scattering properties in the absence of metals (Figure 2, Figure 3 and Figure 4).

Turbidimetry and DLS assays showed that metals induce large aggregates that are turbid to light. Interestingly, the effect of copper is larger for HβA1 than the zinc effect; however, the opposite behavior was found for HβB2, where zinc is more aggressive than copper. These results confirm that each metal has a specific interaction and effect in each of the tested proteins. The addition of EDTA reduces the turbidity and recovers some of the soluble protein, indicating that some of the aggregation is due to metal-mediated bridging [30,43].

We then used florescence to obtain insight into the conformational changes that occur due to the metal interaction. The Trp residues buried within the hydrophobic core allow the core of the protein to be monitored [14,16]; we used ANS fluorescence to monitor the exposure of newly formed hydrophobic patches [44,45]. Interestingly, zinc-induced aggregation for HβA1 showed no change in intrinsic tryptophan fluorescence, but a ~4-fold increase in ANS fluorescence. This fact suggests that the protein core remains folded, but the hydrophobic patches are exposed by metal binding. One possible scenario would be if there was an opening between the two domains of the protein, which would then promote the formation of the observed aggregation.

On the other hand, copper-induced aggregation for HβA1 showed changes in Trp fluorescence and also an increase in ANS, which would be consistent with intermediates that expose hydrophobic patches and a partial unfolding of some domain.

For HβB2, we observe a change in Trp fluorescence, which also indicates some changes in the core of the protein. Unexpectedly, we observed a very large increase in hydrophobic patches for Zn(II), suggesting a much more drastic effect than for copper. These results are in contrast to what has been observed for some other crystallins. The data for HγD show that Zn(II) ion only induces aggregation, without unfolding the protein, mainly by intermolecular metal-ion-bridged aggregates, while copper is the one that produces a partial unfolding of the domains [33,42]. Furthermore, in the aggregation of HγS induced by metal ions, zinc aggregation was promoted mainly by metal bridges, whereas copper-ion-induced aggregation proceeds through a more complex mechanism involving oxidation and destabilization [30].

Interestingly, IR measurements of the two proteins with the metal ions showed no significant changes, suggesting that the metal interaction does not have a large effect on the secondary structure. Similar results have been observed for HγD crystalline, where, although the NMR measurement detects opening of the protein core [33], this unfolding exhibits relatively little alteration in the CD-measured secondary structure [42].

These results show that metal-induced protein aggregation involves different degrees of conformational changes as mechanisms. Therefore, we investigated whether the metal reduces the protein stability. We observed that both metals reduce the thermostability of the proteins.

Our results show that copper and zinc ions specifically induce the aggregation of β-crystallins, some of the more abundant crystallins in the lens implicated in cataract disease. Although both Cu(II) and Zn(II) ions promote aggregation, their effects are very different and involve a complex mixture of destabilization, domain opening, unfolding, and metal bridging. Our work provides insights into the mechanisms of metal-induced aggregation of the crystallins in the human body.

## 4. Materials and Methods

### 4.1. Protein Expression and Purification

Recombinant proteins HβA1 and HβB2 were expressed in BL21-RIL *Escherichia coli* cells transformed with plasmids pE-SUMO CRYBA1 and pE-SUMO CRYBB2, respectively. The plasmid pE-SUMO contains a SUMO tag followed by a His tag. The cultures were grown in LB super broth medium supplemented with 100 μg/mL of ampicillin and 30 μg/mL of chloramphenicol at 37 °C to an optical density at 600 nm of 2.0. Protein production was induced by addition of 0.5 mM isopropyl D-thiogalactoside (IPTG) at 18 °C for 12 h. The cells were harvested by centrifugation at 4000 rpm for 30 min at 4 °C. The pellet was resuspended in 50-mM Tris-HCl pH 8.0 and 10-mM imidazole (buffer A), and then lysed by adding lysozyme for 30 min using 10 cycles of 1 min of sonication. The lysed cells were centrifuged at 14,000 rpm for 45 min at 4 °C.

The resulting supernatant was subsequently subjected to several chromatographic steps: The supernatant was applied to a 5 mL HisTrap affinity column (GE Healthcare). The column was then washed with 15 mL of buffer A. The protein was eluted from the column with a linear gradient of 15 mL imidazole from 10 mM to 500 mM. Protein-containing fractions were determined by SDS-PAGE. The fractions were cleaved by adding SUMO protease Ulp1 (sigma) at 18 °C. Samples were passed through a second 5 mL HisTrap affinity column to remove the His-tagged SUMO protein and protease. Protein fractions were applied on a Q-Sepharose column (Fast Flow 20 mL, GE Healthcare) which was pre-equilibrated with 10 mM Hepes pH 8.0 and 100 mM NaCl (buffer B). The column was then washed with 20 mL of buffer B at a flow rate of 1 mL/min. Proteins were eluted from the column with a 60 mL linear gradient of 100–500 mM NaCl.

### 4.2. UV-Visible Spectroscopy

The effect of Cu(II) or Zn(II) on protein aggregation kinetics was followed by turbidimetry measurements using UV-visible spectroscopy. Data were acquired on an Agilent 8453 UV-visible diode array spectrophotometer (Agilent, Santa Clara, Cal, CA, USA). Protein samples were 10-mM hepes pH 8.0 and 100-mM NaCl. For turbidity assays, the protein was incubated in the presence and absence of 0.5, 1, and 1.5 equivalents of CuSO_4_ or ZnSO_4_ at 37 °C for 2 h. Absorbance changes were measured at 405 nm every 15 s. The measurement variation was estimated from three successive recorded data points and the measurement error was calculated. The experiments were carried out in triplicate for each condition.

The amount of soluble protein was determined in the absence and presence of 1.5 equivalents of CuSO_4_ or ZnSO_4_ and the presence of an excess of EDTA (30 equivalents) at the end of the experiments. The samples were centrifuged at 14,000 rpm and at 20 °C. Solubility was determined by the maximum protein concentration (mg/mL) in the soluble fractions.

### 4.3. Fluorescence

Fluorescence spectra were recorded on a Cary Eclipse spectrophotometer (Agilent, Santa Clara, Cal, CA, USA). The conformational changes of the proteins in the absence and presence of 1.5 equivalents of CuSO_4_ or ZnSO_4_ were followed by Trp fluorescence. Intrinsic fluorescence spectra were recorded at 37 °C. The length of the cuvette path was 1 cm and the protein concentration was 50 µM. The emission spectrum was recorded in the range of 300 and 500 nm using an excitation wavelength of 295 nm.

To calculate the maximum signal emission when the protein was unfolded, spectra were acquired with urea (6 M for HβA1 and 4 M for HβB2) [14,16,46,47]; quantities reported where the proteins are completely unfolded; Figure S6). The obtained values were used to estimate the change in the Trp environment of the protein as a function of protein unfolding.

Emission signals at 344 and 360 nm were used to calculate the 360/344 ratio for HβA1. Emission signals at 332 and 350 nm were used to calculate the 350/332 ratio for HβB2.

The fluorescence of 8-anilinonaphthalene-1-sulfonic acid (ANS) was used as an indicator of the hydrophobic surface exposure of proteins in the absence and presence of 1.5 equivalents of CuSO_4_ or ZnSO_4_. Samples for ANS fluorescence were prepared by equilibrating protein solutions with an excess of ANS (8-fold) for 30 min in the dark.

Samples were prepared at a final protein concentration of 50 µM with 400 µM ANS in buffer B. Extrinsic ANS fluorescence was excited at 380 nm and emission spectra were collected in wavelength ranges from 400 to 600 nm. All spectroscopic experiments were performed at 37 °C.

The stability of the proteins as a function of temperature and urea was studied using samples at a protein concentration of 50 µM, in buffer B in the absence and presence of 1.5 equivalents of CuSO_4_ or ZnSO_4_. Thermal denaturation of proteins was carried out by heating protein solutions continuously from 37 °C to 90 °C. Thermal transition curves were obtained by measuring intrinsic fluorescence of Trp and extrinsic of ANS to monitor structural changes every 2 °C after equilibrium for 2 min at a specified temperature.

Triplicate experiments were performed for each condition.

### 4.4. Fourier Transform Infrared Spectroscopic Analysis

IR absorption spectra were recorded using a NICOLET 6700 spectrometer (Thermo-Electron Corp., Waltham, MA, USA) in the spectral region of 525–4000 cm^−1^. A background spectrum was measured and subtracted. Spectra were processed using the general purpose peak fitting Fityk software [48]. After subtraction of the buffer, the resulting amide I band contours were deconvoluted. Four bands were fitted automatically; then, each component in the amide I band was computed as a fractional area of the corresponding peak divided by the sum of the total area.

The peaks obtained by deconvolution of the amide I band can be assigned to specific types of secondary structure. Components between 1640 and 1660 cm^−1^ are assigned to α-helix; components in the regions of 1620–1640 cm^−1^ and 1670–1695 cm^−1^ (high frequencies) are assigned to β-sheet; components between 1650–1695 cm^−1^ are assignment to β-turns; components between 1640 and 1650 cm^−1^ are usually associated with random coil [39,40].

### 4.5. Dynamic Light Scattering

Changes in the size of the protein samples were measured by DLS. Measurements were made on a Zetasizer Nano ZSP spectrophotometer (Malvern Panalytical, Egham, UK), with a dispersion angle of 173°. Protein samples were 50 µM in buffer B. All samples were centrifuged for 5 min at 9000 rpm and filtered through a 0.22 µm filter prior to measurements. Size changes over time were measured by incubating proteins in the absence and presence of 1.5 equivalents of CuSO_4_ or ZnSO_4_ at 37 °C for 40 min. Three runs with 10 scans of 10 s were obtained for each measured data point. Triplicate experiments were performed for each condition.

Data were analyzed by the distribution methods implemented in the SEDFIT software, which allows adjusting different models [49]. The data were used to obtain translational diffusion coefficients through measurement of the correlation coefficient. Prediction of hydrodynamic radius from the structure was calculated using HULLRAD server [38].

### 4.6. Computational Methods

The amino acid sequence of human crystallins γ and β were taken from Uniprot. Sequence alignments were performed using the ‘Align’ tool [50]; then, the position of residues with propensity to interact with metals was analyzed (His, Cys, Glu, and Asp residues). The alignments are shown in Figure 1 and Figure S2.

Initial coordinates for HβB2 were taken from the crystal structures (PDB ID: 1YTQ). A homology monomer model of HβA1 was generated using the crystal structure of human β-crystallin A4 as a template (PDB ID:3LWK) using SWISS-MODEL [51]. Then, a face-en-face dimer was modeled, taking advantage of the oligomeric structure prediction implemented in the SWISS-MODEL server [52], using the crystal structure dimer of human β-crystallin A4 (PDB ID:3LWK) as template. A homology monomer model of HβA1 was generated using the crystal structure of human β-crystallin A4 as template (PDB ID:3LWK); then, a face-en-face dimer was modeled. For HβB2, the crystal structure of the domain-swapped homodimer of human βB2 crystallin (PDB ID: 1YTQ) was used to produce a dimer. Models were generated using SWISS-MODEL server.

To predict potential metal binding sites, MIB (Metal Ion-Binding Site Prediction) [36] was used. The structures from the dimer models were submitted to the server, selecting Zn and Cu ions. Additionally, we also used BioMetAll software to predict the potential metal-binding site based on the geometric organization of the protein backbone [37]. This software does not distinguish between different metal ions. The results are plotted in Figure S3.

## Figures and Tables

**Table 1:** Soluble protein. HβA1 and HβB2 with and without EDTA.

	Protein Recovered −EDTA	Protein Recovered +EDTA
HβA1	100%	100%
HβA1 + 1.5 eq Zn(II)	18 ± 8%	70 ± 5%
HβA1 + 1.5 eq Cu(II)	18 ± 5%	20 ± 5%
HβB2	100%	100%
HβB2 + 1.5 eq Zn(II)	10 ± 5%	20 ± 5%
HβB2 + 1.5 eq Cu(II)	18 ± 7%	35 ± 5%

## Data Availability

The data that support the findings of this study are available on request from the corresponding author.

## Supplementary Materials

The following supporting information can be downloaded at https://www.mdpi.com/article/10.3390/molecules27092970/s1. Figure S1: Dimer models. Figure S2: Sequence alignment. Figure S3A: Predicted copper binding sites for HβA1 and HβB2 by MIB and BioMetalAll. Figure S3B: Predicted zinc binding sites for HβA1 and HβB2 by MIB and BioMetalAll. Figure S4: Effect of Zn(II) and Cu(II) as reported by the absorbance spectroscopy. Figure S5: Replica of protein oligomerization induced by metal-binding by DLS. Figure S6: Normalized fluorescence spectra of tryptophan after chemical unfolding by urea. Figure S7: Replica of normalized intrinsic fluorescence. Figure S8: IR peak deconvolution of Amide I region and assignment to secondary structure elements. Figure S9: Effect of Cu(II) and Zn(II) ions in the thermal stability of the β-crystallins.
