# Supplementary material for: Zinc and Copper Ions Induce Aggregation of Human β-Crystallins
Source: Molecules. 2022 May 6;27(9):2970. doi: 10.3390/molecules27092970 (PMC9105653; doi:10.3390/molecules27092970)
Supplement: Supplementary file 1 [file molecules-27-02970-s001.zip › molecules-1630097-supplementary.pdf]

molecules

Article

Zinc and Copper Ions Induce Aggregation of Human β-crystallins

Vanesa Ramirez-Bello, Javier Martinez-Seoane, Arline Fernández-Silva, and Carlos Amero*

LABRMN, Centro de Investigaciones Químicas, Instituto de Investigación en Ciencias Básicas y Aplicadas, Universidad Autónoma del Estado de Morelos, Morelos 62209, Mexico

* Correspondence: carlosamero@uaem.mx; Tel.: +52-777-329-7900 ext.6043

1. Supplementary Materials:

- **Figure S1.** Dimer models.
- **Figure S2.** Sequence alignment.
- **Figure S3A.** Predicted copper binding sites.
- **Figure S3B.** Predicted zinc binding sites.
- **Figure S4.** Effect of Zn(II) and Cu(II) as reported by the absorbance spectroscopy.
- **Figure S5.** Replica of protein oligomerization induced by metal-binding by DLS.
- **Figure S6.** Normalized fluorescence spectra of tryptophan after chemical unfolding by urea.
- **Figure S7.** Replica of normalized Intrinsic fluorescence.
- **Figure S8.** IR peak deconvolution of Amide I region and assignment to secondary structure elements.
- **Figure S9.** Effect of Cu(II) and Zn(II) ions in the thermal stability of the β-crystallins.

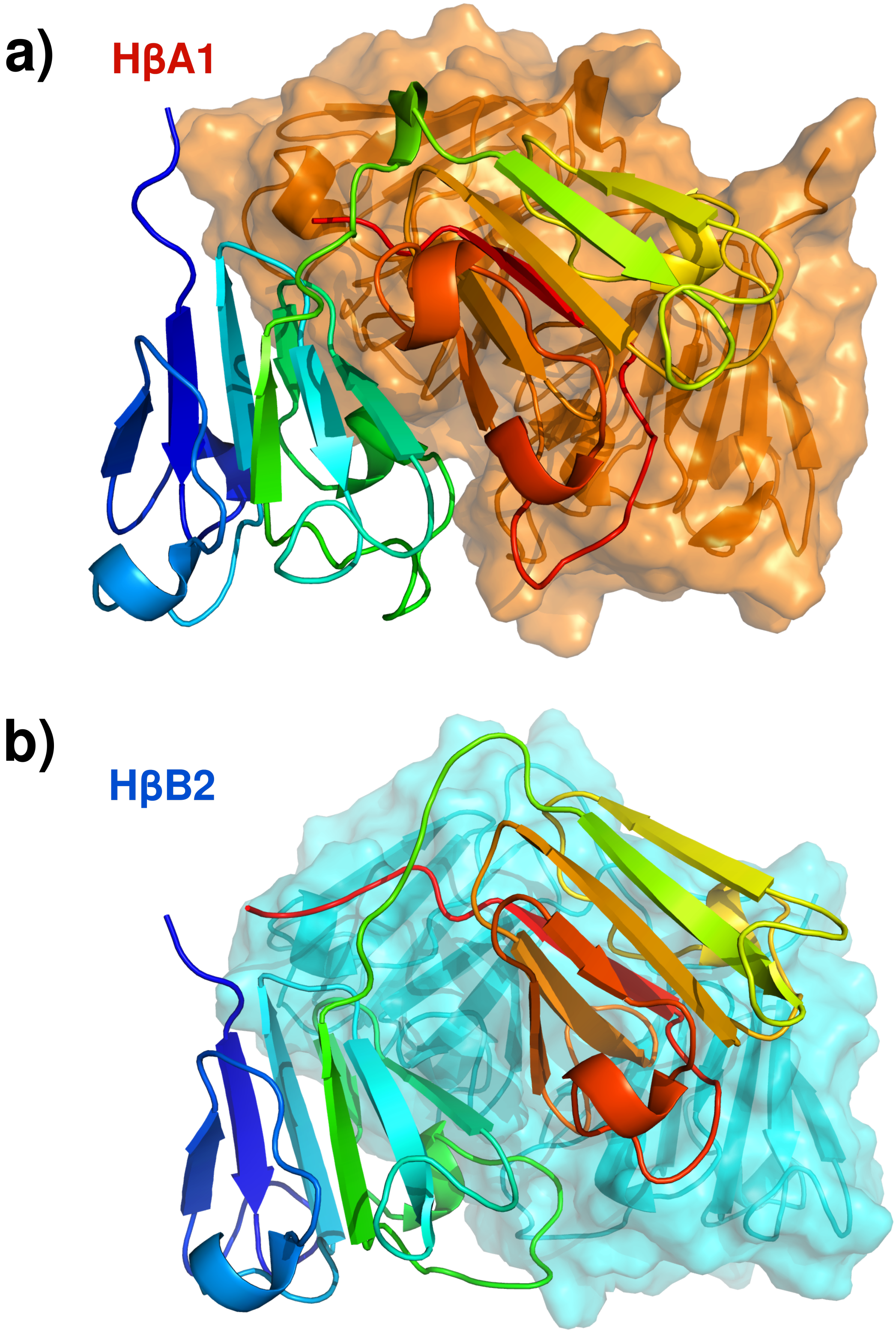

Figure S1. Dimer models. Three-dimensional structure models of face-en-face dimer of a) HβA1 and b) HβB2. Homology monomer model of HβA1 and HβB2 was generated using 3LWK and 1YTQ, respectively. Models were generated using SWISS-MODEL server.

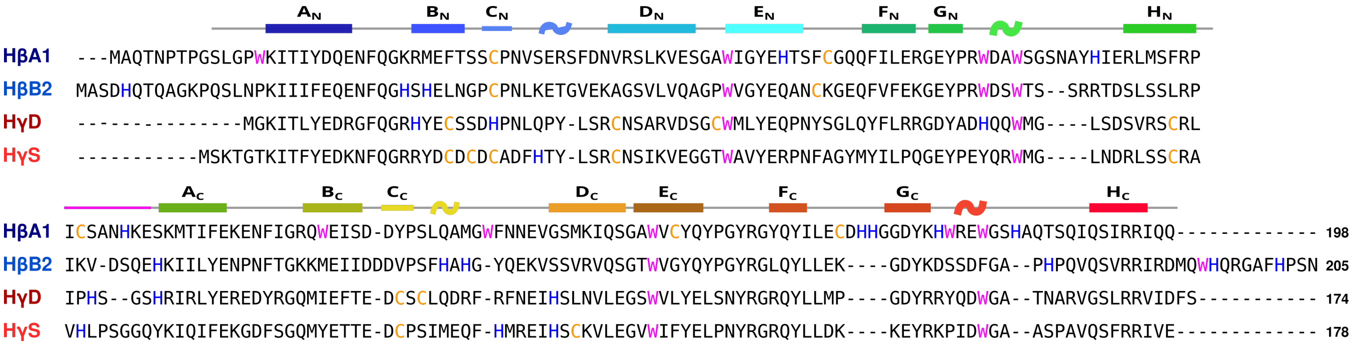

Figure S2. Sequence alignment. Sequence alignment of HγD, HγS HβA1 and HβB2 crystallins. The elements of the secondary structure of the N-terminal and C-terminal domains and the linker region are shown. Histidine residues are shown in blue, cysteines in yellow, and tryptophan in magenta. The alignment of the β/γ-crystallins revealed that, although several features are conserved in the proteins, the reported binding sites for gamma C, D and S are not conserved in the beta crystallins. Histidines and cysteines residues are the most common binding residues for copper and zinc.

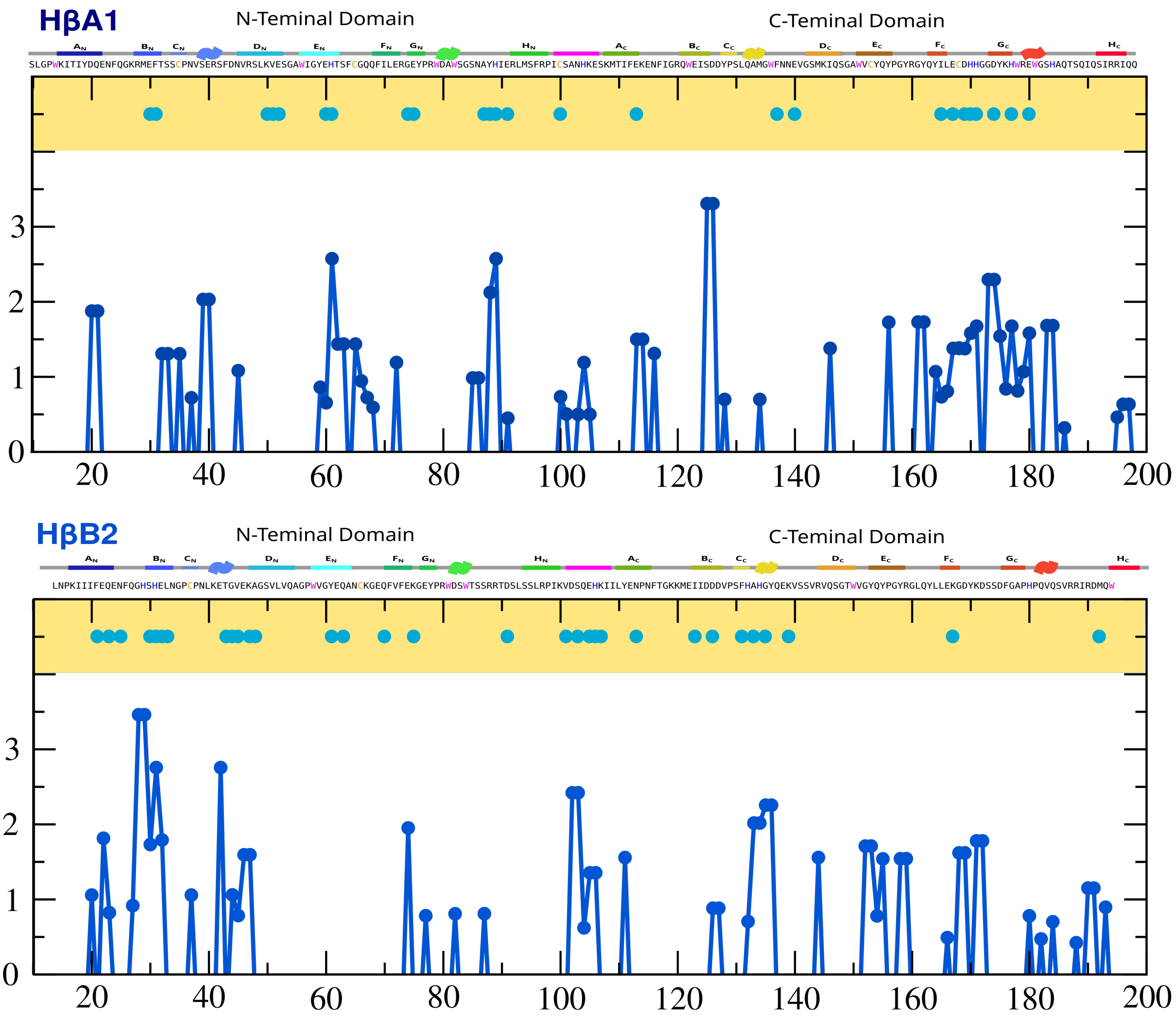

Figure S3A. Predicted copper binding sites. For HβA1 and HβB2 by MIB (blue lines) and BioMetalAll (Top: Yellow background with blue circles). MIB searches for sequence similarities between the target protein and previously reported metal-binding site proteins, while BioMetAll looks for characteristic sequences in the protein backbone.

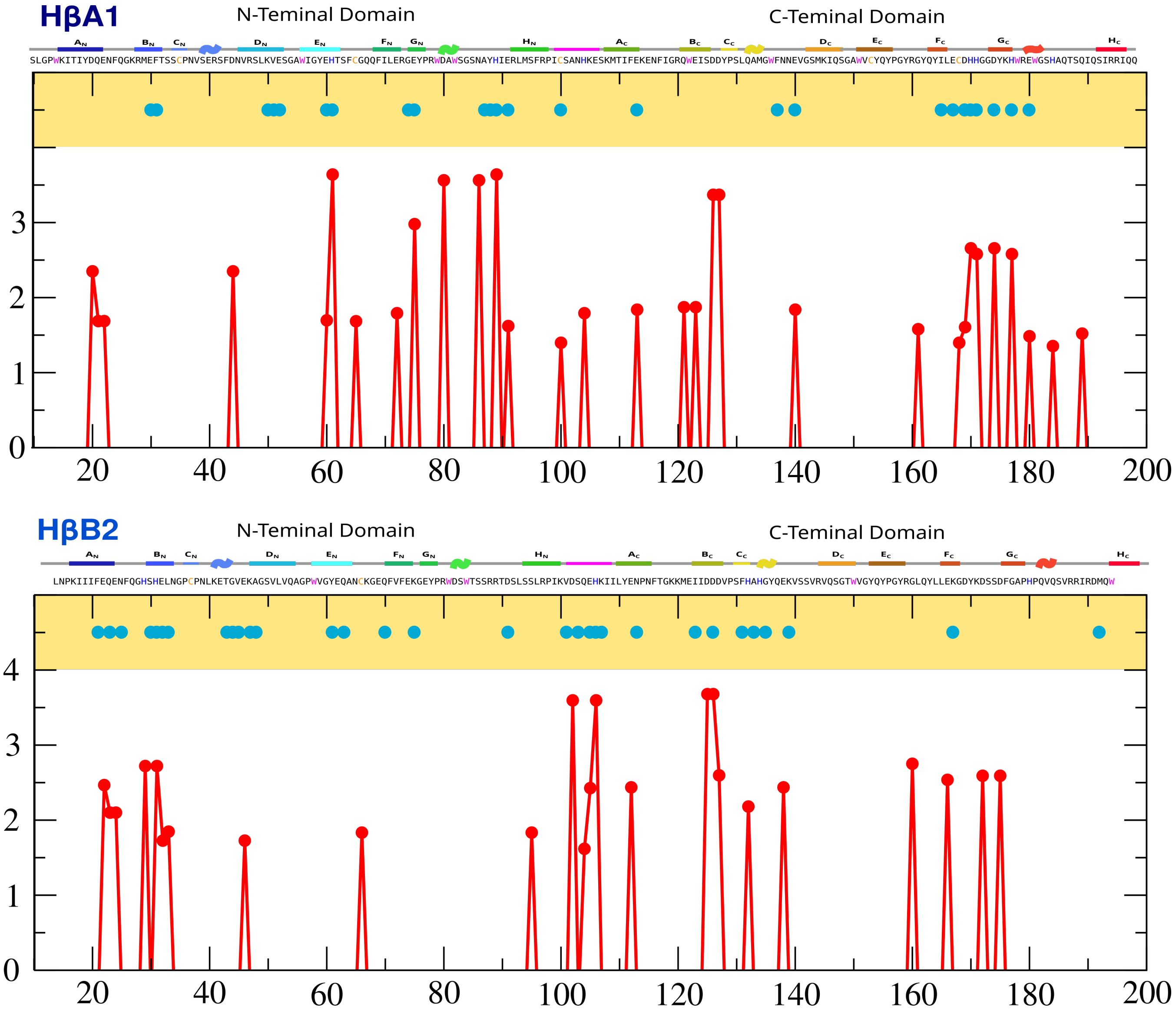

Figure S3B. Predicted zinc binding sites. For HβA1 and HβB2 by MIB (red lines) and BioMetalAll (Top: Yellow background with blue circles).

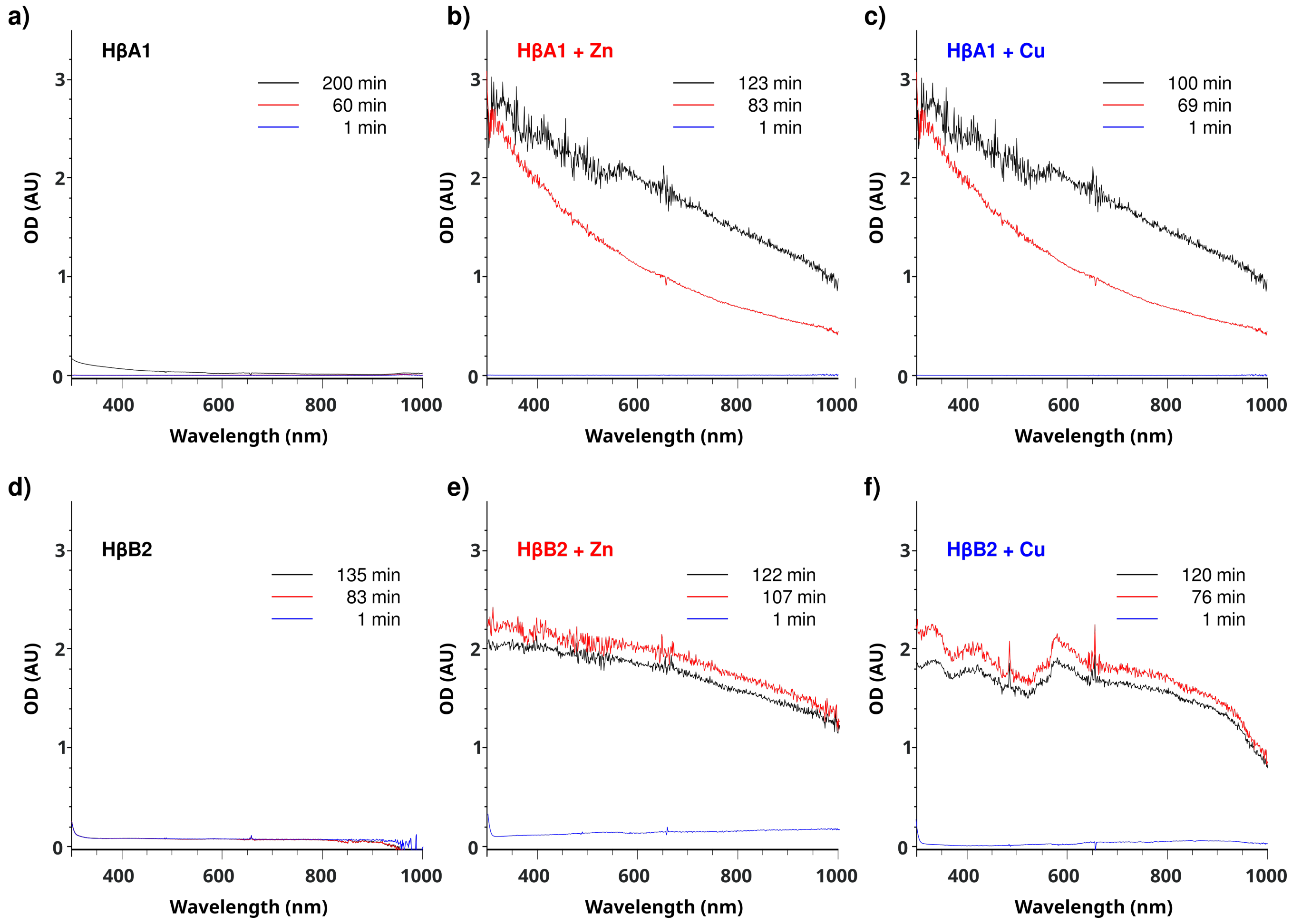

Figure S4. Effect of Zn(II) and Cu(II) as reported by the absorbance spectroscopy. a) HβA1 at 37°C after different periods of time. b) HβA1 at 37°C in the presence of 1.5 equivalents of Zn(II) after different periods of time. c) HβA1 at 37°C in the presence of 1.5 equivalents of Cu(II) after different periods of time. d) HβB2 at 37°C after different periods of time. e) HβB2 at 37°C in the presence of 1.5 equivalents of Zn(II) after different periods of time. f) HβB2 at 37°C in the presence of 1.5 equivalents of Cu(II) after different periods of time. A spectrum is shown at the beginning (blue), one at the middle (red), and one at the end (black). The change in absorbance is due to the formation of aggregates that scatter the light.

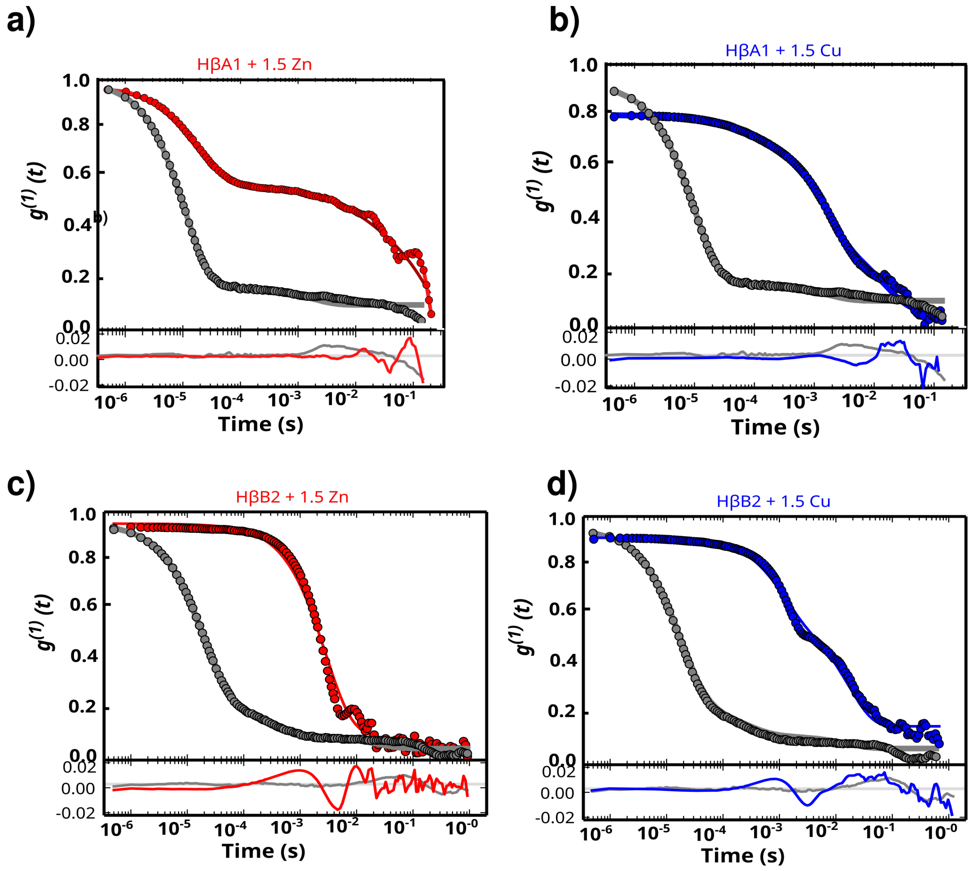

Figure S5. Replica of protein oligomerization induced by metal-binding by DLS. a) HβA1 correlation coefficient in the absence (gray) and presence of 1.5 equivalents of Zn(II) (red). b) HβA1 correlation coefficient in the absence (gray) and presence of 1.5 equivalents of Cu(II) (blue). c) HβB2 correlation coefficient in the absence (gray) and presence of 1.5 equivalents of Zn(II) (red). d) HβB2 correlation coefficient in the absence (gray) and presence of 1.5 equivalents of Cu(II) (blue). Measurements with metal ions yielded a shift to the right indicating an increase in size due to oligomerization.

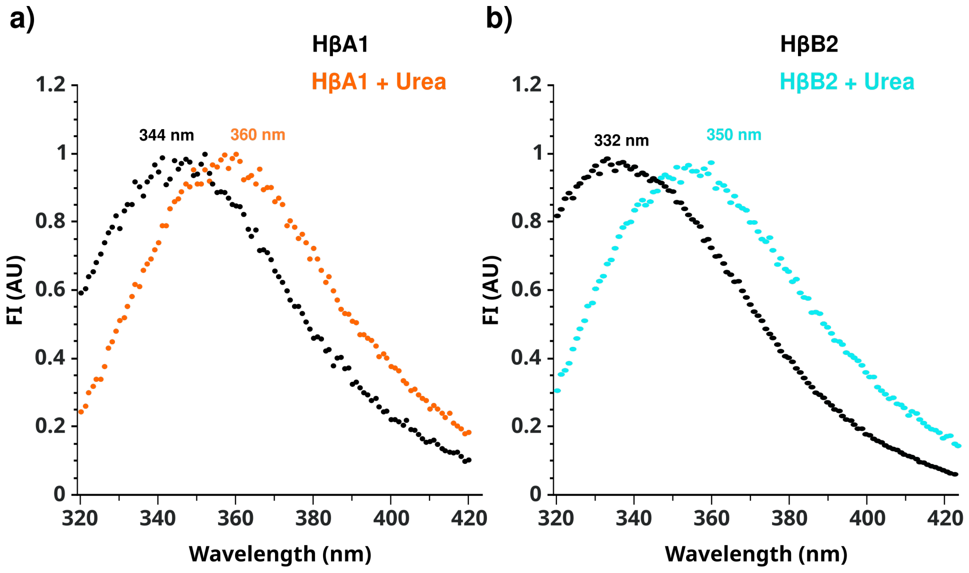

Figure S6. Normalized fluorescence spectra of tryptophan after chemical unfolding by urea. a) HβA1 folded (black) and HβA1 + 6M Urea (orange). b) HβB2 folded (black) and HβB2 + 4M Urea (cyan). For HβA1, the emission maximum for the intrinsic fluorescence of the native protein is around 344 nm, while the maximum shifts to 360 nm when the protein unfolds. The maximum emission for the intrinsic fluorescence of the native HβB2 is 332 nm, while this maximum shifts to 350 nm when the HβB2 is fully unfolded.

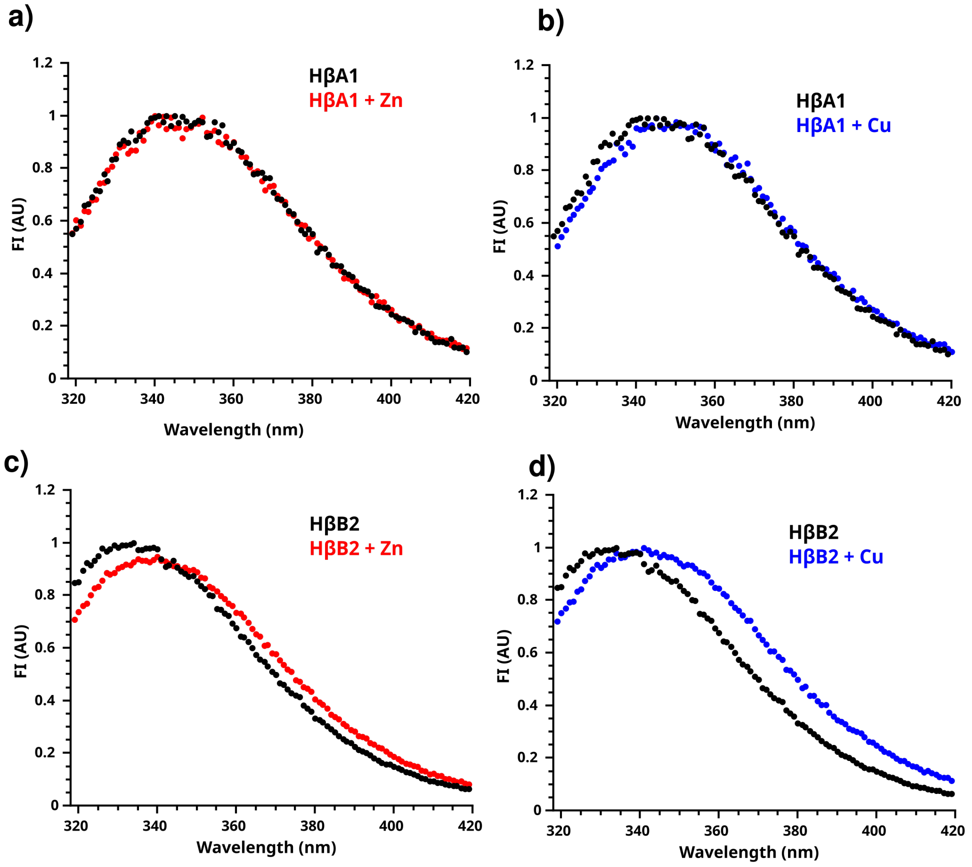

Figure S7. Replica of normalized Intrinsic fluorescence. a) HβA1 in the absence of metal ions (black) and in the presence of Zn(II) (red). b) HβA1 in the absence of metal ions (black) and in the presence of Cu(II) (blue). c) HβB2 in the absence of metal ions (black) and in the presence of Zn(II) (red). d) HβB2 in the absence of metal ions (black) and in the presence of Cu(II) (blue). The emission spectrum was recorded in the range of 300 and 500 nm using an excitation wavelength of 295 nm at 37 °C.

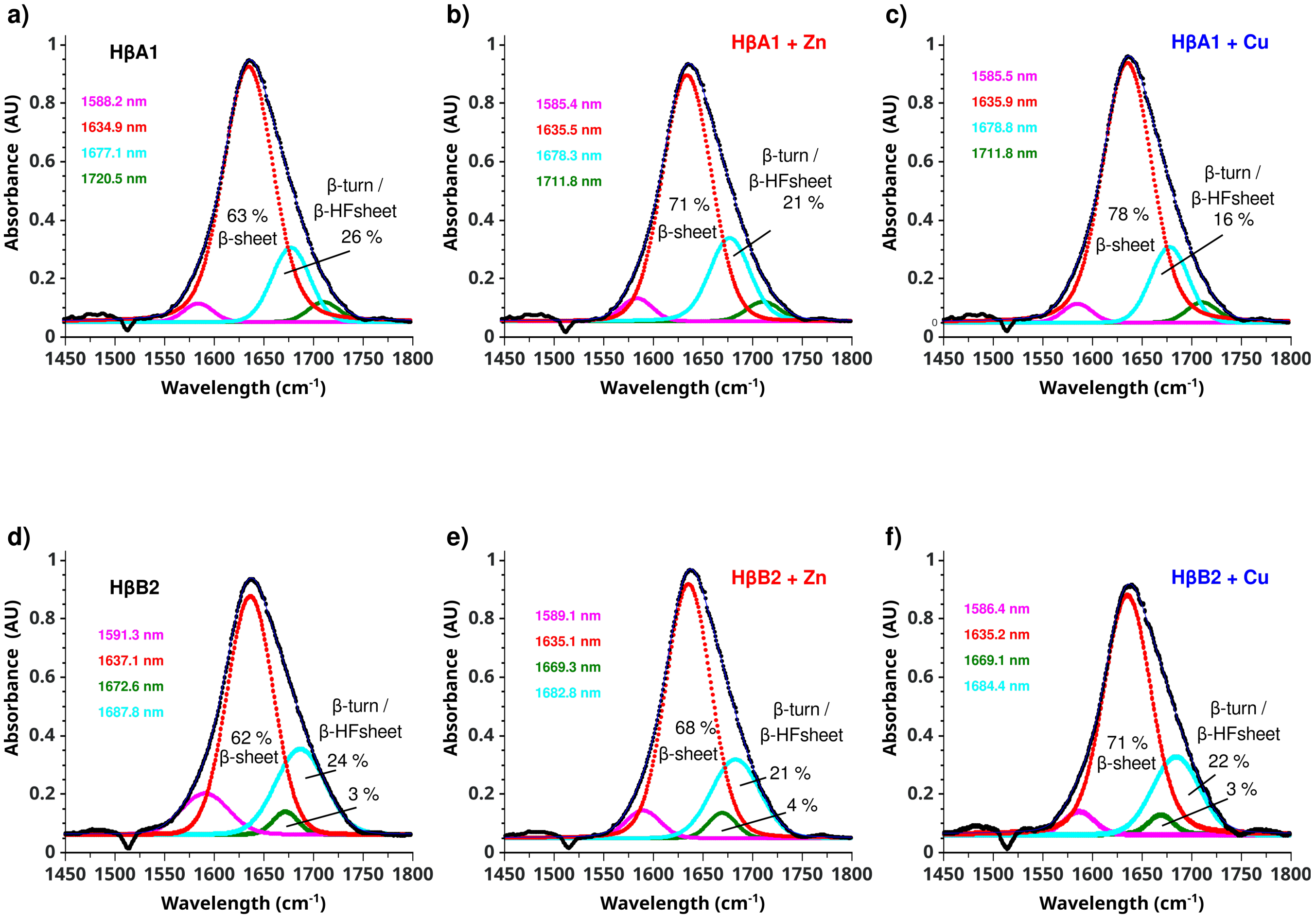

Figure S8. IR peak deconvolution of Amide I region and assignment to secondary structure elements. a) Amide I band of the FTIR spectrum of HβA1. b) Amide I band of the FTIR spectrum of HβA1 in the presence of Zn(II). c) Amide I band of the FTIR spectrum of HβA1 in the presence of Cu(II). d) Amide I band of the FTIR spectrum of HβB2. e) Amide I band of the FTIR spectrum of HβB2 in the presence of Zn(II). f) Amide I band of the FTIR spectrum of HβB2 in the presence of Cu(II). Original spectra are represented by black circles. The blue line is the fitted contour due to the addition of the four deconvoluted peaks.

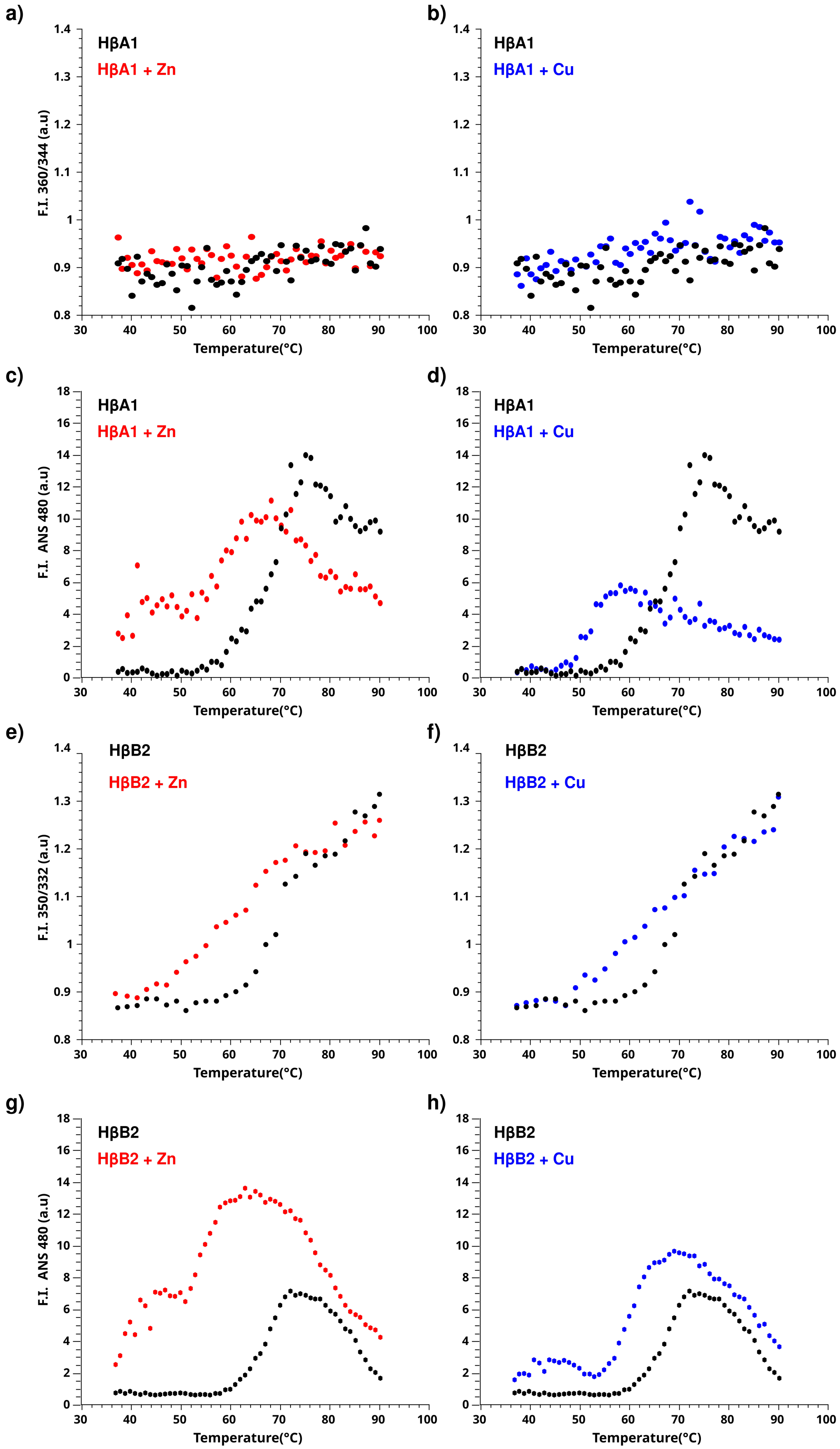
a)
HβA1
HβA1 + Zn
F.I. 360/344 (a.u)
Temperature(°C)
b)
HβA1
HβA1 + Cu
Temperature(°C)
c)
HβA1
HβA1 + Zn
F.I. ANS 480 (a.u)
Temperature(°C)
d)
HβA1
HβA1 + Cu
Temperature(°C)
e)
HβB2
HβB2 + Zn
F.I. 350/332 (a.u)
Temperature(°C)
f)
HβB2
HβB2 + Cu
Temperature(°C)
g)
HβB2
HβB2 + Zn
F.I. ANS 480 (a.u)
Temperature(°C)
h)
HβB2
HβB2 + Cu
Temperature(°C)

Figure S9. Effect of Cu(II) and Zn(II) ions in the thermal stability of the β-crystallins. Denaturation experiments were performed from 37 to 90°C u sing intrinsic Trp fluorescence and extrinsic ANS fluorescence. Thermal stability of the HβA1 without the metals and with 1.5 eq of Cu(II) and Zn(II) monitored using (a,b,e,f) tryptophan fluorescence and (c,d,g,h): ANS fluorescence at 480 nm. Overall, these results show that the interaction of Zn(II) and Cu(II) ions with both beta crystallins decreases their thermal stability.
